# Dynamic m^6^A Methylation in Neurodevelopmental and Neurodegenerative Disorders

**DOI:** 10.3390/epigenomes10040062

**Published:** 2026-10-03

**Authors:** Ambrose Loc Ngo, Xuemei Qu, Niki Gharavi Alkhansari, Linda Nguyen, Uyen Tam Nguyen, Huiping Zhang

**Affiliations:** 1 College of Osteopathic Medicine, Kansas City University, Farber-McIntire Campus, 2901 St. Johns Blvd, Joplin, MO 64804-1598, USA; ambroseloc.ngo@kansascity.edu (A.L.N.); niki.gharavi@kansascity.edu (N.G.A.); uyentam.nguyen@kansascity.edu (U.T.N.); 2 The Human Physiology Program, Sargent College of Health & Rehabilitation Sciences, Boston University, 635 Commonwealth Avenue, Boston, MA 02215-1610, USA; aleciaqu@bu.edu; 3 College of Pharmacy, Western University, California Campus, 309 E. Second St., Pomona, CA 91766-1854, USA; linda.nguyen2@westernu.edu; 4 Department of Psychiatry, Boston University Chobanian & Avedisian School of Medicine, 72 East Concord Street, Boston, MA 02118-2526, USA; 5 The Biomedical Genetics Section, Department of Medicine, Boston University Chobanian & Avedisian School of Medicine, 72 East Concord Street, Boston, MA 02118-2526, USA

**Keywords:** m^6^A RNA modification, m^6^A writers, m^6^A erasers, m^6^A readers, neurodevelopmental disorders, neurodegenerative diseases

## Abstract

N^6^-methyladenosine (m^6^A), the most prevalent internal modification in eukaryotic RNA, has emerged as a key epitranscriptomic regulator in the central nervous system, coordinating gene expression programs that govern neural development, synaptic plasticity, and neuronal adaptation throughout the lifespan. This review synthesizes emerging evidence that m^6^A dysregulation represents a shared epitranscriptomic mechanism linking neurodevelopmental and neurodegenerative disorders, highlighting a mechanistic convergence that bridges these traditionally distinct disease categories. Dysregulation of m^6^A-mediated control of neurogenesis, synaptic maturation, and dopaminergic development has been implicated in neurodevelopmental disorders, including autism spectrum disorder, attention-deficit/hyperactivity disorder, and intellectual disability. In neurodegenerative diseases such as Alzheimer’s disease, Parkinson’s disease, and Huntington’s disease, m^6^A dysregulation mediates multiple interconnected pathogenic processes, including neuroinflammation, mitochondrial dysfunction, tau hyperphosphorylation and aggregation, α-synuclein dysregulation, and impaired neuronal repair. Environmental stressors and metabolic perturbations modulate m^6^A modification patterns, suggesting that gene–environment interactions contribute to disease susceptibility through epitranscriptomic mechanisms. m^6^A modification functions as a dynamic molecular switch governing neural plasticity and circuit function across early development, adulthood, and aging, and its dysregulation predisposes individuals to neurological disease manifestation throughout the lifespan. Advances in cell-type-specific and circuit-resolved m^6^A profiling will deepen our understanding of m^6^A dysregulation and strengthen its translational potential as both a diagnostic biomarker and a therapeutic target in neurological diseases. Furthermore, we evaluate pharmacological and molecular strategies targeting m^6^A regulatory machinery, including the writers METTL3/METTL14, the erasers FTO/ALKBH5, and reader proteins, as promising therapeutic approaches to restore aberrant neuronal circuit function in neurodevelopmental and neurodegenerative disorders.

## 1. Introduction

The regulation of gene expression in the nervous system extends beyond DNA sequence and chromatin architecture to include post-transcriptional modifications of RNA, collectively referred to as the epitranscriptome. Among more than 150 identified RNA modifications, N^6^-methyladenosine (m^6^A) is the most abundant internal modification of eukaryotic messenger RNA and decorates thousands of transcripts across diverse cell types [1,2]. The advent of transcriptome-wide m^6^A profiling technologies has revealed RNA methylation as a dynamic and information-rich regulatory layer, reshaping traditional models of gene regulation in complex tissues such as the brain. m^6^A modification is regulated by conserved and reversible machinery comprising methyltransferases (‘writers’), demethylases (‘erasers’), and RNA-binding proteins (‘readers’) that collectively influence RNA splicing, stability, localization, and translation efficiency [1,2]. Yang et al. demonstrated that m^6^A modification affects nearly every stage of mRNA metabolism, establishing it as a versatile post-transcriptional regulatory signal capable of tuning gene expression in response to cellular context [2].

The reversibility of m^6^A distinguishes it from static genetic or chromatin-based modifications and enables temporal flexibility that is particularly well-suited to the dynamic regulatory demands of neural systems. Neurons require precise spatiotemporal control of RNA metabolism to support synaptogenesis, dendritic spine remodeling, axonal growth, and activity-dependent plasticity. Accumulating evidence indicates that m^6^A-modified transcripts are enriched in neuronal genes and preferentially localized to dendrites, axons, and synapses, where local translation supports circuit refinement and plasticity [3]. Within this framework, m^6^A functions as a molecular switch, linking neuronal activity to selective control of transcript fate.

The functional importance of this epitranscriptomic regulation has been demonstrated in vivo. A study showed that genetic deletion of the m^6^A reader YTHDF1 (YTH domain-containing family protein 1) in mice leads to impaired hippocampus-dependent learning, reduced synaptic transmission, and defective long-term potentiation [4]. These deficits were reversible by hippocampal re-expression of YTHDF1, while acute disruption of YTHDF1 or the m^6^A writer METTL3 (N^6^-adenosine methyltransferase 3) in the adult hippocampus recapitulated the synaptic and behavioral phenotypes. Mechanistically, m^6^A recognition by YTHDF1 promotes activity-dependent translation of synaptic plasticity-related transcripts, establishing m^6^A as a direct molecular driver of learning-related protein synthesis.

Despite growing evidence of shared molecular mechanisms across brain disorders, neurodevelopmental and neurodegenerative diseases have largely been studied in isolation, limiting opportunities to identify common therapeutic targets. Studying neurodevelopmental and neurodegenerative disorders in isolation risks overlooking two important possibilities: that early-life disruptions in RNA metabolism may confer lasting vulnerability to neurodegeneration, and that age-related epitranscriptomic alterations may reactivate maladaptive developmental programs later in life. Recent evidence implicates m^6^A dysregulation as a convergent mechanism across the lifespan. Castro-Hernández et al. reported a conserved reduction in m^6^A RNA modifications during aging across multiple neurodegeneration models, with preferential loss of m^6^A on synaptic transcripts critical for neuronal communication and plasticity [5]. Notably, these changes were observed across species and disease models, suggesting that age-related decline in m^6^A-dependent synaptic regulation may represent a conserved molecular signature linking normal aging to neurodegenerative pathology.

**Figure 1:**
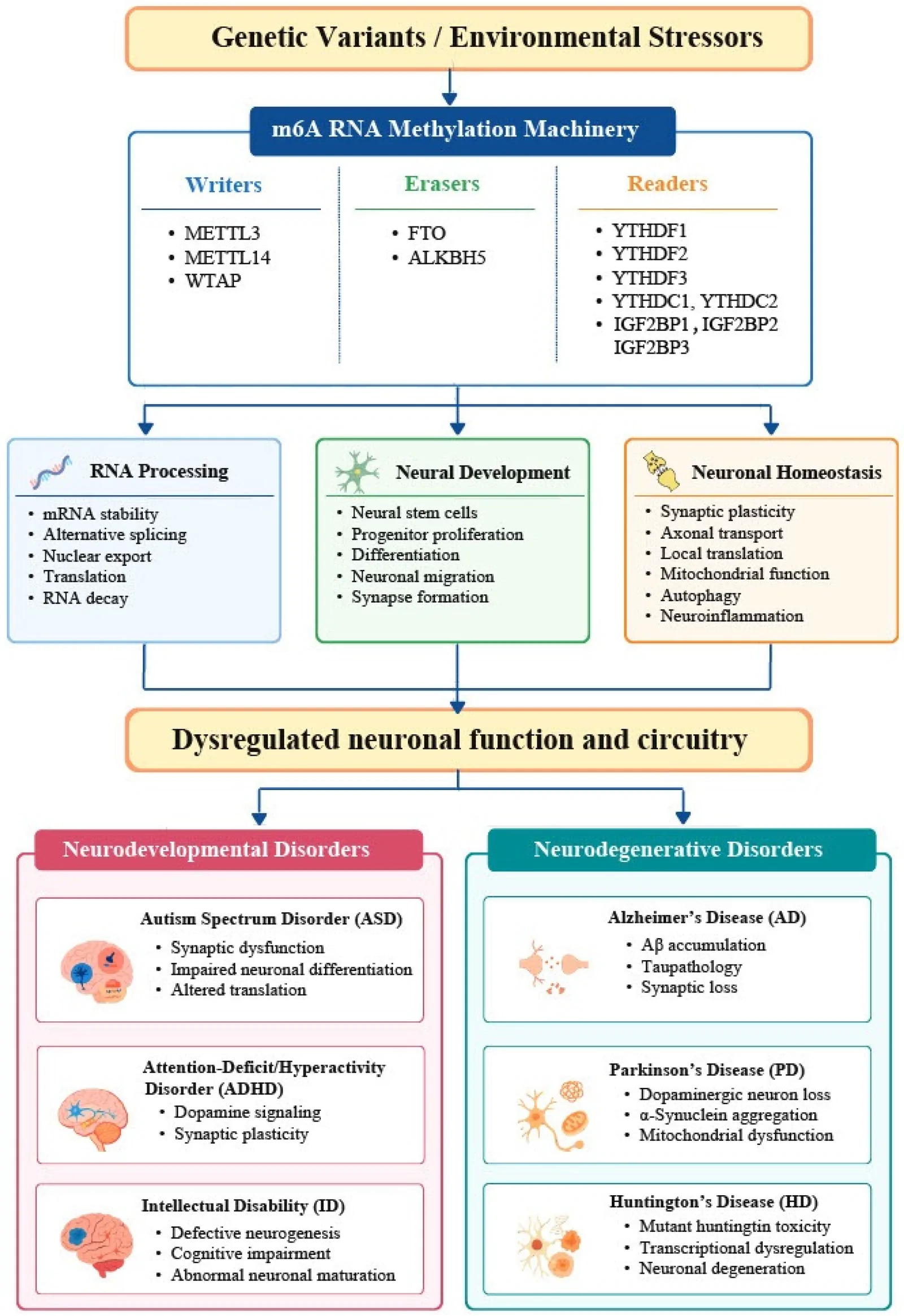
Role of m^6^A RNA methylation in neurodevelopmental and neurodegenerative disorders. m^6^A RNA methylation is dynamically regulated by methyltransferases (“writers”), demethylases (“erasers”), and m^6^A-binding proteins (“readers”). These regulators control multiple aspects of RNA metabolism, including mRNA stability, splicing, export, translation, and decay, thereby influencing neural progenitor proliferation, neuronal differentiation, migration, and synaptic maturation. Dysregulation of m^6^A signaling disrupts neuronal circuit formation and contributes to the pathogenesis of neurodevelopmental and neurodegenerative disorders.

Taken together, these findings position m^6^A as a regulatory axis spanning early neurodevelopment, adult neural plasticity, and aging-related neurodegeneration. In this review, we adopt a lifespan perspective to examine how m^6^A RNA methylation influences brain health and contributes to neurological disease across early development, adulthood, and aging. We synthesize current evidence on the roles of m^6^A methylation in neurodevelopmental and neurodegenerative diseases, integrating findings across neuronal and glial cell types, environmental exposures, and epigenetic crosstalk. By bridging these traditionally distinct research paradigms, we aim to highlight m^6^A as a central epitranscriptomic regulator of neural plasticity, vulnerability, and resilience (Figure 1).

## 2. m^6^A RNA Methylation Machinery and Regulation of Lifespan

The m^6^A modification system comprises three functional components: writers, erasers, and reader proteins. Writers install m^6^A marks through the catalytic activity of the METTL3-METTL14 methyltransferase complex, in which METTL3 is the catalytic subunit, METTL14 contributes to RNA substrate recognition and complex stability, and Wilms tumor 1-associated protein (WTAP) facilitates complex assembly and localization. Erasers remove m^6^A through demethylase activity; the two characterized erasers are fat mass and obesity-associated protein (FTO) and α-ketoglutarate-dependent dioxygenase alkB homolog 5 (ALKBH5). Readers recognize m^6^A-modified RNA and recruit downstream effectors that determine mRNA fate. Key readers include the YTH-domain proteins YTHDF1, YTHDF2, and YTHDF3, which are predominantly localized in the cytoplasm, and YTHDC1 and YTHDC2, which are primarily localized in the nucleus and cytoplasm, respectively; these proteins directly recognize m^6^A through their YTH domains. In addition, the IGF2 mRNA-binding proteins IGF2BP1, IGF2BP2, and IGF2BP3, which are predominantly cytoplasmic, recognize m^6^A-containing RNAs through their RNA-binding domains, whereas heterogeneous nuclear ribonucleoproteins (HNRNPA2B1, HNRNPC, and HNRNPG), which are primarily nuclear, recognize RNA features associated with m^6^A, including m^6^A-induced changes in RNA structure. Collectively, this reversible system dynamically regulates mRNA metabolism including stability, translation efficiency, and subcellular localization [6].

m^6^A writer, eraser, and reader activity is dynamically regulated across neurodevelopmental stages, controlling critical neurogenic processes. METTL14 and FTO direct neuronal proliferation and differentiation; their genetic ablation produces distinct developmental phenotypes. Embryonic *Mettl14*-knockout mice exhibit reduced neurogenesis and delayed differentiation of cortical neuron subtypes [7], whereas postnatal *Mettl3*- and *Alkbh5*-knockout mice show disrupted glial maturation and impaired dendritic arborization [8]. Similarly, YTHDF1 knockdown in human glioblastoma cells suppresses cell migration and proliferation [9], highlighting m^6^A-reader-dependent control of developmental and pathogenic processes.

Across the brain’s lifespan, m^6^A methylation demonstrates ever-changing dynamics. In the embryonic brain, increased m^6^A activity impacts neural stem cell proliferation and differentiation. m^6^A writers and erasers regulate oncogenic signaling cascades; their upregulation promotes aberrant proliferation of neural progenitors and differentiated neurons, contributing to gliomagenesis and neuronal malignancy [10]. During the postnatal stage, m^6^A methylation serves to regulate cerebellar development through additional neuronal cell proliferation and differentiation, as well as improve function in RNA splicing, transport, and translation [8]. m^6^A machinery is essential for brain development, regulating mRNA stability, translation efficiency, and localization to control neurogenesis, neuronal differentiation, and synaptic maturation [11]. As m^6^A levels increase in adult brains compared to embryonic brains, it is speculated that m^6^A plays a role in regulating more complex brain functions [12,13]. In the adult brain, the function of m^6^A shifts towards regulation of mature neurons. The m^6^A machinery is responsible for the transportation and translation of mRNA, which is crucial for the maintenance of synapses and neuronal plasticity [14]. In an aging brain, dysfunction in m^6^A activity contributes to neurodegeneration and cognitive decline. *Mettl3* deletion impairs memory consolidation in mice, underscoring the critical role of m^6^A methylation in long-term memory formation [15]. A link has also been established between m^6^A modifications and Alzheimer’s disease. When comparing the makeup of RNA-seq data in individuals with Alzheimer’s disease to normal individuals, there is a decrease in expression of synapse-related genes. m^6^A methylation levels are dysregulated in individuals with Alzheimer’s disease compared to healthy controls. Specifically, m^6^A modification regulates microtubule-associated protein tau (MAPT) pre-mRNA splicing and stability, controlling tau protein expression; dysregulation of this m^6^A-dependent control can lead to pathological tau accumulation and aggregation, hallmarks of Alzheimer’s disease [16].

A comprehensive literature search of PubMed, Web of Science, and Scopus (2016–2026) identified peer-reviewed articles examining m^6^A methylation in neurodevelopmental and neurodegenerative diseases. Search terms included combinations of: (‘m^6^A methylation’ OR ‘N^6^-methyladenosine’) AND (‘neurodevelopmental disorder*’ OR ‘autism spectrum disorder’ OR ‘attention-deficit/hyperactivity disorder’ OR ‘intellectual disability’ OR ‘Rett syndrome’ OR ‘developmental delay’) AND (‘neurodegenerative disease*’ OR ‘Alzheimer’s disease’ OR ‘Parkinson’s disease’ OR ‘Huntington’s disease’). Inclusion criteria were: (1) peer-reviewed original research or comprehensive reviews, (2) studies in human subjects, animal models, or cell culture systems, (3) direct mechanistic examination of m^6^A modification in neurodevelopmental or neurodegenerative pathology, and (4) English-language publications. Studies were excluded if they: (1) presented review commentary without novel data, (2) focused exclusively on non-neural tissues, or (3) were opinion pieces or editorials. Title and abstract screening was performed independently, and full texts of potentially relevant articles were retrieved and evaluated against inclusion criteria.

## 3. m^6^A Methylation and Neurodevelopmental Disorders

m^6^A RNA methylation is critical for neurodevelopment, modulating neural stem cell proliferation, cortical neurogenesis, synaptogenesis, and circuit maturation. Dysregulation of these processes has been linked to neurodevelopmental disorders such as autism spectrum disorder (ASD), attention-deficit/hyperactivity disorder (ADHD), and intellectual disability (Table 1). For example, the m^6^A “writers” METTL3 and METTL14 control the timing of neurogenesis by regulating the balance between proliferation and differentiation of neural progenitor cells; conditional loss of these enzymes in mouse models produces abnormal cortical layering and impaired neuronal differentiation, highlighting m^6^A’s role in cortical development [17]. Similarly, the m^6^A “reader” YTHDF2 promotes the degradation of methylated transcripts to refine gene expression programs during cortical neurodevelopment, indicating that precise control of transcript stability is required for proper developmental timing [18].

### 3.1. m^6^A RNA Methylation in Autism Spectrum Disorder (ASD)

Accumulating evidence implicates m^6^A RNA methylation as a key epitranscriptomic regulator of processes frequently disrupted in autism spectrum disorder (ASD), including progenitor proliferation, cortical lamination, axon guidance, synaptogenesis, and dendritic spine maturation. m^6^A “writers,” “readers,” and “erasers” modulate transcript stability, localization, and translation to control the timing and fidelity of neurodevelopmental gene programs; perturbation of this regulation can produce circuit- and behavior-level deficits relevant to ASD.

Functional studies in animal models further illustrate these principles. Conditional loss of core m^6^A writer components, such as METTL3 or METTL14, or readers such as YTHDF2, results in abnormal cortical layering, impaired neuronal differentiation, reduced axonal branching, and disorganized neural circuits; these phenotypes mirror aspects of ASD-associated neuropathology [13,19,21,42]. Likewise, fragile X mental retardation protein (FMRP), an RNA-binding protein that interacts with m^6^A-modified transcripts, facilitates the nuclear export of transcripts, including microtubule-associated protein 1B (MAP1B) and β-III-tubulin precursor mRNAs, that are required for neuronal differentiation. Loss of FMRP function leads to nuclear retention of these transcripts, impaired progenitor cell-cycle progression, and abnormal dendritic spine morphology, features associated with Fragile X syndrome and related neurodevelopmental phenotypes [22,23,24].

m^6^A also directly influences synaptic function. YTHDF1 promotes translation of synaptic proteins (for example, Robo3.1) by recruiting translation initiation machinery and stabilizing a closed-loop mRNA configuration [20,21,25,43,44]. *Ythdf1* knockout mice exhibit reduced hippocampal synaptic transmission, impaired learning, repetitive behaviors, and striatal dysfunction; these deficits are rescued by *Ythdf1* re-expression [4,21,26]. These findings align with work showing that dysregulated translation initiation (e.g., elevated eIF4E activity) produces ASD-like phenotypes that can be mitigated by pharmacologic modulation of translation initiation complexes [45,46,47].

Transcriptome-wide and human genetic studies further support the relevance of m^6^A regulation to ASD. m^6^A-modified transcripts, including those encoding key neurodevelopmental transcription factors such as PAX6, SOX1, SOX2, EMX2, and NEUROG2 (Neurogenin 2), are enriched in neurodevelopmental pathways and ASD-associated gene sets in human and mouse forebrain tissues [17,48]. Human genetic studies have also identified components of the m^6^A regulatory machinery, including YTHDC2 in East Asian cohorts, as candidate ASD-associated genes, suggesting a potential link between genetic variation in m^6^A-related pathways and ASD risk [27]. Together, these molecular, cellular, transcriptomic, and genetic findings suggest that dysregulation of m^6^A-mediated RNA processing may alter neurodevelopmental timing and synaptic function and thereby contribute to ASD-related phenotypes.

### 3.2. m^6^A RNA Methylation in Attention-Deficit/Hyperactivity Disorder (ADHD)

Emerging evidence implicates m^6^A RNA methylation, particularly FTO-mediated demethylation, in the pathophysiology of attention-deficit/hyperactivity disorder (ADHD). Genetic association studies and animal models have linked *FTO* variants to attention deficits, impulsivity, and broader neurodevelopmental syndromes characterized by behavioral dysregulation. For example, common *FTO* polymorphisms such as rs9939609 have been associated with increased ADHD risk in several cohorts [28,29], and targeted manipulation of *Fto* in rodents alters locomotor activity, reward sensitivity, and attentional performance [30].

Mechanistically, FTO regulates m^6^A marks on mRNAs that control neuronal differentiation, synaptic formation, and circuit refinement during development. In the midbrain and hippocampus, i.e., the regions important for attention, working memory, and executive control, FTO activity modulates the stability and translational efficiency of transcripts encoding synaptic proteins, ion channels, and components of dopamine signaling pathways [30,31]. Key FTO target mRNAs include *Drd2* (dopamine receptor D2), *Slc6a3* (dopamine transporter, DAT), *Snap25* (synaptosomal-associated protein 25), and *Th* (tyrosine hydroxylase). FTO-mediated m^6^A removal on *Drd2* mRNA increases its stability and translational efficiency, elevating D2 receptor surface expression on medium spiny neurons; conversely, targeted deletion of *Fto* in D2-expressing medium spiny neurons increases m^6^A occupancy on *Drd2* mRNA, reduces D2 receptor levels, and measurably alters locomotor reactivity, attentional set-shifting, and reward sensitivity in behavioral paradigms directly relevant to ADHD [30]. FTO similarly demethylates m^6^A on *Snap25* mRNA; reduced SNAP-25 protein following FTO perturbation impairs presynaptic vesicle fusion and dopamine release kinetics, contributing to the hypodopaminergic state that underlies attentional and impulse-control deficits [31]. Furthermore, hypermethylation of *Th* mRNA in *FTO*-deficient dopaminergic neurons reduces tyrosine hydroxylase expression, dampening dopamine biosynthesis and reinforcing the dopaminergic deficit. Together, these transcript-specific *FTO*-m^6^A interactions provide a direct molecular mechanism through which genetic variation in *FTO* shapes dopaminergic circuit function and ADHD symptomatology.

At the cellular level, altered m^6^A dynamics can affect dendritic spine maturation and synaptic plasticity, leading to deficits in information processing and cognitive flexibility. At the systems level, such changes may disrupt frontostriatal and frontoparietal networks that subserve attention and inhibitory control [5,32,33]. In rodent studies, *Fto* knockdown or overexpression leads to measurable changes in synaptic transmission and behavioral paradigms relevant to ADHD [34], supporting a causal link between m^6^A regulation and attentional phenotypes.

Importantly, m^6^A-mediated regulation is context- and cell type-specific: the consequences of FTO perturbation differ between excitatory and inhibitory neurons and across developmental windows [49]. This specificity may help explain heterogeneity in ADHD presentation and treatment response. Although the detailed molecular cascades connecting *FTO* variants to human ADHD remain to be fully delineated, converging genetic, molecular, and functional data support a model in which dysregulated m^6^A methylation contributes to the development and maintenance of attention and impulse-control deficits. Further studies integrating human genetics, cell-type-resolved epitranscriptomics, and circuit-level functional assays are needed to clarify the therapeutic potential of targeting m^6^A pathways in ADHD.

### 3.3. m^6^A RNA Methylation in Other Neurodevelopmental Disorders

Beyond ASD and ADHD, m^6^A RNA methylation has been implicated in several other neurodevelopmental disorders, including intellectual disability, neurodevelopmental delay, and syndromic conditions such as Rett syndrome and certain forms of epileptic encephalopathy.

m^6^A RNA methylation has emerged as a key contributor to intellectual disability by regulating gene expression programs essential for neurodevelopment. Mutations or dysregulated expression of m^6^A writers (e.g., METTL5), erasers (ALKBH5, FTO), and readers (YTH family) have been identified in cohorts with intellectual disability and produce defects in neural progenitor proliferation, neuronal differentiation, and synaptic maturation in cellular and animal models [50]. Notably, pathogenic variants in *METTL5*, which encodes an 18S rRNA m^6^A methyltransferase, cause autosomal recessive intellectual disability and microcephaly [36,51], highlighting the importance of m^6^A-mediated RNA regulation in brain development. Collectively, these findings indicate that m^6^A dysregulation can generate heterogeneous cognitive phenotypes, positioning the m^6^A pathway as a critical mechanistic axis and a promising therapeutic target in intellectual disabilities.

m^6^A RNA methylation regulates post-transcriptional programs essential for early brain maturation, and perturbations in this pathway impair neurodevelopmental trajectories. Loss-of-function or altered expression of m^6^A writers (METTL3, METTL14), erasers (ALKBH5, FTO), or readers (YTH proteins) disrupt neural progenitor proliferation, neuronal differentiation, and migration in both in vitro and in vivo models [18,37,52,53]. For example, embryos from YTHDF2-deficient mothers exhibit developmental delay in both mouse and zebrafish models, demonstrating the maternal contribution of m^6^A reader function to neurodevelopment [18,37]. Mechanistically, m^6^A dysregulation alters the stability, splicing, and localized translation of specific transcripts encoding cytoskeletal proteins, synaptic factors, and metabolic enzymes that are indispensable for normal brain maturation [18]. For cytoskeletal transcripts, impaired m^6^A regulation of *MAP1B*, *TUBB3*, and *DCLK1* mRNAs reduces microtubule-associated protein availability at developing growth cones, compromising axon guidance, neuronal migration, and dendritic branching. For synaptic transcripts, dysregulated m^6^A modification of *DLG4*/*PSD-95*, *SHANK3*, *SYNGAP1*, and *BDNF* mRNAs impairs activity-dependent local translation at nascent synapses, reducing post-synaptic scaffold assembly, disrupting glutamatergic receptor clustering, and attenuating BDNF-mediated synaptic strengthening. For metabolic transcripts, aberrant m^6^A methylation of *GLUL*, *GLS*, and *LDHA* mRNAs compromises glutamate biosynthesis and glycolytic energy production, limiting the metabolic capacity required to sustain synaptogenesis and circuit refinement. Together, these transcript-specific alterations impair dendritic growth, reduce synaptogenesis, and disrupt neural circuit assembly. By coordinately controlling the stability and translational efficiency of these developmentally critical transcripts, m^6^A modification represents a key mechanistic hub in neurodevelopmental processes and a compelling target for investigating the molecular etiology of developmental delay and developing novel therapeutic strategies.

Rett syndrome, a neurodevelopmental disorder most often caused by mutations in *MECP2*, shows links to m^6^A RNA methylation through convergent effects on neuronal maturation and synaptic function [38]. Dysregulation of m^6^A writers, erasers, or readers alters the stability, splicing, and translation of MECP2-regulated transcripts, including those governing dendritic arborization, synaptic scaffolding, and activity-dependent plasticity. Because m^6^A regulation is highly cell-type and developmental-stage specific, dysregulated m^6^A signaling may modulate the severity and progression of Rett phenotypes and represents a complementary epitranscriptomic axis for mechanistic study and potential therapeutic exploration.

m^6^A RNA methylation has been implicated in epilepsy through its regulation of transcripts that control neuronal excitability, synaptic development, and network stability. In cellular and animal models, disruption of m^6^A machinery is associated with abnormal neuronal firing patterns, impaired synaptic maturation, and seizure-like phenotypes, linking epitranscriptomic dysregulation to the pathophysiology of severe, early-onset epileptic disorders [39,40,41,54]. These findings suggest m^6^A pathways may modulate seizure susceptibility and represent a potential target for mechanistic studies and therapeutic development.

### 3.4. Summary of m^6^A RNA Methylation and Neurodevelopmental Disorders

Collectively, these data indicate that aberrant m^6^A-mediated post-transcriptional control is a convergent mechanism that can perturb neurodevelopmental programs and lead to cognitive, motor, and epileptic phenotypes, making the m^6^A pathway a promising focus for mechanistic investigation and potential therapeutic targeting.

## 4. m^6^A Methylation and Neurodegenerative Diseases

m^6^A methylation plays a critical role in regulating neuronal homeostasis and is increasingly implicated in neurodegenerative disease pathogenesis. Dysregulation of m^6^A methylation machinery, including alterations in writer, eraser, and reader protein function, has been linked to multiple pathological hallmarks of neurodegeneration: disrupted proteostasis, impaired autophagy, mitochondrial dysfunction, and aberrant RNA metabolism [55,56,57,58]. These molecular disturbances are observed across major neurodegenerative diseases, including Alzheimer’s disease (AD), Parkinson’s disease (PD), and Huntington’s disease (HD). Mounting evidence suggests that m^6^A dysregulation directly contributes to the accumulation of pathogenic proteins, defective neuroinflammatory responses, and accelerated neuronal degeneration, positioning m^6^A modification as a promising and therapeutically actionable target in these devastating conditions (Table 2).

### 4.1. m^6^A RNA Methylation in Alzheimer’s Disease (AD)

Accumulating evidence indicates that dysregulation of m^6^A methylation is a central pathological mechanism in Alzheimer’s disease (AD). Multiple studies report significant alterations in m^6^A writers (METTL3, METTL14), erasers (FTO), and reader proteins in AD brain tissue, with profound functional consequences for disease-relevant signaling pathways [26,59,80]. Reduced m^6^A levels have been documented in hippocampal and cortical pyramidal neurons from both AD patients and transgenic AD models, correlating with decreased METTL3 expression in memory-critical regions including the hippocampus and middle temporal gyrus [60,61]. This m^6^A hypomethylation directly contributes to impaired synaptic integrity and defective memory consolidation. Supporting this mechanism, *METTL3* knockdown in mice induces a spectrum of AD-relevant pathological features: memory deficits, reduced synaptic density, tau hyperphosphorylation, and increased susceptibility to amyloid-β (Aβ)-induced synaptic damage. Notably, these detrimental phenotypes are rescued by METTL3 overexpression, providing compelling evidence for the neuroprotective role of m^6^A methylation in maintaining cognitive function and neuronal homeostasis [61].

Mechanistically, m^6^A dysregulation contributes to multiple pathological hallmarks of AD. FTO influences mTOR signaling and tau phosphorylation, while METTL3 directly localizes to both Aβ plaques and neurofibrillary tangles [60,62,81]. Aβ stimulation suppresses METTL3 expression in neurons, leading to oxidative stress, synaptic protein loss (e.g., PSD95), and activation of apoptotic pathways; conversely, FTO overexpression enhances tau phosphorylation via mTOR activation, whereas METTL3 promotes autophagic clearance of phosphorylated tau [61]. The m^6^A reader protein HNRNPA2B1 further exacerbates neurotoxicity by translocating from the nucleus to the cytoplasm during tau oligomerization, facilitating interaction between m^6^A-tagged transcripts and tau aggregates [63]. METTL3 loss also triggers aberrant cell-cycle reentry in post-mitotic neurons, driving oxidative stress, neuroinflammation, and apoptosis, i.e., key contributors to AD pathology [61].

Spatial and temporal aspects of m^6^A dysregulation add another layer of complexity. In the 5×FAD mouse model, m^6^A methylation is locally reduced near Aβ plaques despite stable global brain m^6^A levels, with early reductions in METTL3 expression followed by WTAP loss at later stages [64]. Notably, dysregulation of m^6^A methylation manifests as early as three months of age in these models, substantially preceding the onset of cognitive symptoms. This temporal relationship suggests that localized m^6^A dysregulation functions as an upstream, initiating molecular event in the Aβ-driven pathogenic cascade [64].

Beyond neuronal dysfunction, m^6^A methylation plays a critical cell-type-specific role in neuroimmune signaling. While METTL3 expression is diminished in neurons, it is upregulated in activated microglia, where it promotes neuroinflammatory signaling via the TRAF6/NF-κB pathway [20]. MeRIP-seq and RNA-seq analyses revealed a redistribution of m^6^A peaks on transcripts enriched in immune, inflammatory, and senescence-associated pathways [65,66]. Notably, m^6^A levels positively correlate with expression of disease-associated microglia (DAM) genes (*Trem2*, *Cst7*, *Cd74*), suggesting that m^6^A modifications regulate microglial activation states [35,82]. Elevated ALKBH5 expression in microglia from AD mouse brains, particularly in regions proximal to Aβ plaques, indicates that m^6^A dysregulation may exacerbate neuroinflammation and cognitive decline by modulating transcriptional programs within microglial populations [67,83].

Transcriptomic and functional studies further illuminate the molecular landscape of m^6^A dysregulation in AD. Integrated transcriptomic analyses identified 42 m^6^A-related differentially expressed genes in postmortem Alzheimer’s disease brain tissue [68]. These genes were enriched for pathways implicated in mitochondrial dysfunction, retinoic acid signaling, and DNA damage repair. Key hub genes included *SOX2*, *KLF4*, *CD44*, *YAP1*, and *AQP1*, which are predominantly expressed in hippocampal and white matter regions selectively vulnerable to AD pathology. Regional colocalization suggests that altered m^6^A methylation patterns coordinate dysregulation of neuroplasticity, apoptotic signaling, and inflammatory responses in susceptible brain compartments. Additionally, m^6^A methylation is essential for maintaining neurovascular unit (NVU) integrity and promoting angiogenesis. Dysregulation of m^6^A writers (METTL3) and erasers (FTO) impairs the expression of critical vascular growth factors, including VEGF and HDGF, leading to compromised blood–brain barrier function, reduced neurogenesis, and impaired glymphatic clearance [69,84].

Collectively, these findings position m^6^A methylation as a multifaceted pathogenic driver in AD, offering promising therapeutic targets for intervention across neuronal, glial, and vascular compartments.

### 4.2. m^6^A Methylation in Parkinson’s Disease (PD)

m^6^A RNA methylation is increasingly recognized as a critical regulator of dopaminergic neuron survival and function in Parkinson’s disease (PD), a neurodegenerative disorder characterized by selective loss of dopaminergic neurons in the substantia nigra and pathological accumulation of α-synuclein in Lewy bodies. m^6^A modifications control key aspects of RNA metabolism, including transcript stability, splicing, nuclear export, and translation, and are particularly abundant in the central nervous system, where they maintain neuronal homeostasis and support regenerative capacity [84,85]. Converging evidence demonstrates dysregulation of m^6^A regulatory machinery in PD. Conditional deletion of the m^6^A writer METTL14 in the substantia nigra reduces tyrosine hydroxylase expression and impairs dopaminergic signaling [70,71,86], while loss of the m^6^A eraser FTO triggers axonal degeneration and compromises dopaminergic neuron viability [72]. Additionally, downregulation of the m^6^A reader HNRNPC attenuates dopaminergic neuron proliferation [73,87], collectively highlighting m^6^A’s essential role in dopaminergic neuron integrity.

Mechanistic studies reveal that global m^6^A levels are reduced in PD cellular and animal models, accompanied by upregulation of demethylases (FTO, ALKBH5) and downregulation of writers (METTL3, METTL14) and readers (YTHDF2, IGF2BP2) [72]. Elevated FTO activity destabilizes neuroprotective transcripts including GRIN1 and ATM, enhancing excitotoxic vulnerability, while reciprocal loss of writer and reader function impairs protective regulatory mechanisms and promotes dysregulation of pathological transcripts such as SNCA (coding for α-synuclein) [74,75,88,89].

Recent machine learning analysis identified 11 m^6^A-related genes with differential expression in PD patients, four of which (*YTHDC2*, *HNRNPC*, *LRPPRC*, and *IGFBP3*) emerged as robust diagnostic candidates [76]. YTHDC2 enhances translation efficiency and mRNA stability; HNRNPC mediates m^6^A-dependent mRNA splicing; LRPPRC regulates mitochondrial function and neurodegeneration; and IGFBP3 modulates neuronal apoptosis and immune signaling. Unsupervised clustering of m^6^A gene expression signatures delineated two PD subtypes with distinct immune infiltration patterns [76], suggesting that m^6^A dysregulation contributes to both cell-autonomous neuronal injury and immune-mediated neurodegeneration.

Overall, m^6^A methylation is dysregulated in PD, with reduced global m^6^A levels and impaired expression of protective genes that maintain dopaminergic neuron survival. Thus, m^6^A-related genes are potential diagnostic biomarkers and therapeutic targets for PD.

### 4.3. m^6^A Methylation in Huntington’s Disease (HD)

Huntington’s disease (HD) is a progressive neurodegenerative disorder characterized by motor dysfunction, cognitive decline, and selective striatal neurodegeneration. Emerging evidence implicates dysregulation of m^6^A RNA methylation in HD pathogenesis, particularly through effects on synaptic plasticity, alternative splicing, and neuronal gene expression.

Studies in the Hdh^+/Q111^ HD mouse model revealed altered m^6^A methylation patterns in the hippocampus, with both pre-symptomatic and symptomatic animals showing hypermethylation of HD-related genes including *Htt*, *Grin2b*, and *Ntrk2*, which are critical for synaptic transmission and plasticity [77,78]. In contrast to wild-type controls, which exhibit dynamic m^6^A remodeling during cognitive training, HD mice display a markedly attenuated m^6^A response characterized by impaired demethylation of learning-associated transcripts. This defect correlates with dysregulated expression of the m^6^A demethylase FTO and the writer METTL14, which fail to respond to training stimuli appropriately in HD mice. Notably, targeted FTO knockdown in hippocampal CA1 restored m^6^A levels and improved memory performance, establishing a causal link between m^6^A dysregulation and cognitive impairment [77,78].

Complementary studies in the R6/2 HD mouse model demonstrated region- and gene-specific m^6^A dysregulation [79]. Reduced METTL3 expression in the striatum and cortex led to widespread decreases in nuclear m^6^A deposition. Single-nucleotide m^6^A eCLIP-seq revealed that genes critical for striatal identity and synaptic function, normally enriched for m^6^A methylation, showed markedly reduced m^6^A tagging in disease tissue. Importantly, this m^6^A deficit impaired TDP-43 binding, an RNA-binding protein that recognizes m^6^A-marked transcripts to regulate alternative splicing. Decreased m^6^A at TDP-43 motifs resulted in exon skipping in long-intron transcripts enriched for neuronal development genes [79]. These findings, corroborated in mouse and human HD tissue, indicate that m^6^A-TDP-43 cooperation maintains post-transcriptional integrity in neurons; dysregulation of this axis contributes to selective vulnerability of striatal and cortical neurons in HD.

Together, m^6^A methylation is dysregulated in Huntington’s disease through impaired remodeling in response to cognitive stimuli, leading to hippocampal dysfunction and cognitive decline. Additionally, reduced m^6^A writer expression in the striatum and cortex disrupts the m^6^A-TDP-43 axis, impairing alternative splicing of genes critical for striatal neuron identity and contributing to selective neurodegeneration.

### 4.4. Summary of m^6^A RNA Methylation and Neurodegenerative Diseases

Collectively, dysregulation of m^6^A methylation has emerged as a critical factor in neurodegenerative disease pathogenesis across Alzheimer’s disease (AD), Parkinson’s disease (PD), and Huntington’s disease (HD). In AD, altered m^6^A patterns dysregulate genes involved in mitochondrial function, DNA repair, and neuroinflammation, with hub genes including *SOX2*, *KLF4*, and *CD44*. In PD, reduced global m^6^A levels, accompanied by upregulation of demethylases (FTO, ALKBH5) and downregulation of writers and readers, impair dopamine synthesis and promote α-synuclein accumulation. In HD, m^6^A dysregulation disrupts coordinated pathways in the hippocampus and striatum. Hippocampal m^6^A dysregulation impairs learning and memory consolidation, while striatal m^6^A-dependent TDP-43 function is compromised, resulting in aberrant splicing of neuronally enriched transcripts and synaptic dysfunction. These region-specific yet mechanistically linked alterations position m^6^A regulatory enzymes as tractable therapeutic targets and potential diagnostic biomarkers for HD and related neurodegenerative diseases.

## 5. m^6^A Regulation in Neural Cell Types: From Neurodevelopment to Neurodegeneration

m^6^A RNA methylation regulates critical functions across distinct neural cell types that collectively maintain brain homeostasis from development through aging. The coordinated dysregulation across multiple brain cell types (neurons, astrocytes, microglia, and oligodendrocytes) amplifies pathogenic cascades. There is also evidence that cell-type-selective restoration of m^6^A function may represent a promising therapeutic strategy for neurodevelopmental and neurodegenerative diseases.

### 5.1. m^6^A Regulation in Neuronal Circuit Assembly: Dendritic Spines, Axon Guidance, and Synaptic Function

Neurons require precise spatiotemporal control of RNA metabolism to support dendritic spine morphogenesis, axon guidance, and synaptic plasticity. m^6^A RNA methylation has emerged as a central regulator of these processes through its control of transcript stability, subcellular localization, and translation efficiency of synaptic and cytoskeletal genes. Transcriptome-wide profiling reveals that m^6^A-modified mRNAs are significantly enriched for genes encoding neuronal differentiation factors, axon-guidance molecules, and synaptic signaling proteins [3,90], indicating that epitranscriptomic regulation is tightly coupled to neuronal morphology and circuit connectivity.

m^6^A-dependent local translation in dendrites and synapses is particularly critical for activity-dependent spine remodeling and synaptic strengthening. The m^6^A reader protein YTHDF1 promotes translation of m^6^A-marked synaptic transcripts in response to neuronal activity, while disruption of m^6^A writers or readers impairs synaptic transmission and long-term potentiation [4]. Beyond synaptic plasticity, m^6^A regulates developmental neuronal wiring through modulation of cytoskeletal regulators and axon-guidance transcripts [7,91].

Collectively, these findings establish neuronal m^6^A signaling as a critical interface between transcriptional programs and activity-dependent local translation, enabling neurons to dynamically remodel their structure and function in response to developmental and experiential signals.

### 5.2. Astrocytic m^6^A Regulation of Neurotransmitter Homeostasis and Metabolic Support

Astrocytes serve critical functions in regulating neurotransmitter recycling, metabolic coupling, and synaptic homeostasis, acting as essential intermediaries in neuron-glia communication. Although astrocyte-specific m^6^A studies remain limited compared to neuronal investigations, emerging evidence indicates that epitranscriptomic mechanisms regulate astrocytic gene expression programs involved in energy metabolism, gliotransmission, and synaptic support [92,93,94]. Recent transcriptomic analyses demonstrate that m^6^A regulatory machinery is dynamically expressed in astrocytes and controls glial gene networks linked to metabolic and synaptic functions [95], suggesting that m^6^A-dependent regulation contributes to astrocytic functional plasticity.

Given astrocytes’ essential role in maintaining extracellular glutamate balance and preventing excitotoxicity, dysregulation of m^6^A-dependent RNA processing may have significant consequences for synaptic stability. In neurodevelopmental contexts, impaired astrocytic support can exacerbate excitatory-inhibitory imbalance, while in aging and neurodegenerative disease, astrocytic m^6^A dysfunction contributes to reduced neuroprotection and increased neuronal vulnerability [5].

Together, these observations establish astrocytic m^6^A signaling as a potentially critical but underexplored regulator of neuron-glia interactions across the lifespan, with implications for understanding astrocyte-dependent pathology in neurodegenerative disease.

### 5.3. Microglial m^6^A Signaling in Synaptic Pruning and Neuroinflammation

Microglia perform essential functions in synaptic pruning during neurodevelopment and in immune surveillance and inflammatory responses in the mature brain. Although direct, microglia-specific investigations of m^6^A regulation in developmental synaptic pruning remain limited, emerging evidence indicates that epitranscriptomic mechanisms influence microglial activation states and inflammatory gene expression. m^6^A writers, readers, and erasers regulate the stability and translation of immune-related transcripts, controlling cytokine production and inflammatory signaling pathways [96,97,98,99]. Moreover, Zhang et al. demonstrated that the m^6^A machinery modulates microglial inflammatory responses in models of ischemic brain injury, showing that altered m^6^A signaling influences pro-inflammatory gene expression and microglial activation [100]. These findings establish m^6^A as a functional regulator of microglial inflammatory states and suggest that similar mechanisms may operate during developmental synaptic refinement.

During development, precisely regulated microglial synaptic pruning is critical for normal circuit maturation; aberrant pruning has been implicated in neurodevelopmental disorders including autism spectrum disorder and schizophrenia. In aging and neurodegenerative disease, sustained microglial activation contributes to chronic neuroinflammation and synaptic loss. Age-associated reductions in m^6^A levels and altered regulation of synaptic transcripts have been observed across neurodegenerative contexts [101], suggesting that disruption of epitranscriptomic homeostasis may indirectly influence microglial-synaptic interactions.

Together, these findings support a model in which m^6^A signaling contributes to microglial functional plasticity across the lifespan, while highlighting the need for future cell-type-specific studies addressing developmental pruning mechanisms.

### 5.4. Oligodendrocyte m^6^A Regulation in Myelination and Remyelination

Oligodendrocyte maturation and myelin production depend on dynamic, precisely coordinated transcriptional programs controlling the expression of myelin-associated genes, lipid biosynthetic enzymes, and cytoskeletal regulators essential for myelin sheath biogenesis, maintenance, and repair. m^6^A RNA methylation has emerged as a critical regulator of oligodendrocyte lineage progression and myelination. Using oligodendrocyte-lineage-specific *Mettl3*/*Mettl14* conditional knockouts, Xu et al. demonstrated that loss of m^6^A methylation impairs oligodendrocyte differentiation and myelin formation, resulting in hypomyelination with reduced expression of myelin structural proteins (MBP, PLP1) and compromised white matter integrity [102]. m^6^A signaling mechanistically modulates the stability and processing of transcripts involved in lipid biosynthesis, cytoskeletal dynamics, and myelin protein assembly—pathways fundamental to efficient myelin formation. Loss of proper m^6^A regulation alters the expression and splicing of oligodendrocyte-specific transcripts, disrupting the temporal coordination required for developmental myelination [102].

These findings have direct implications for neurodevelopmental disorders characterized by white matter abnormalities, including autism spectrum disorder and intellectual disability, in which altered myelination and oligodendrocyte dysfunction are increasingly implicated. In the aging and diseased brain, m^6^A-dependent regulation of oligodendrocyte function influences remyelination capacity, which declines with age and is impaired in neurodegenerative and demyelinating conditions.

Together, these observations establish dysregulated oligodendrocyte m^6^A signaling as a shared molecular mechanism linking developmental myelination deficits with impaired remyelination capacity in neurodegeneration.

## 6. m^6^A in Gene–Environment Interactions: From Neurodevelopment to Neurodegeneration

Environmental exposures shape brain development and aging by modulating gene regulatory programs that integrate external signals with cellular state. m^6^A RNA methylation is dynamic, reversible, and responsive to metabolic and stress-related cues, positioning m^6^A machinery as a critical interface between environmental perturbations and post-transcriptional gene regulation in the nervous system.

### 6.1. Prenatal Exposures and m^6^A Disruption in Neurodevelopmental Disorders

Prenatal and perinatal hypoxia are well-established environmental risk factors for altered neurodevelopment by disrupting transcriptional and metabolic programs in neural progenitors. The m^6^A demethylases FTO and ALKBH5 are Fe(II)/α-ketoglutarate-dependent dioxygenases, rendering m^6^A regulation intrinsically sensitive to oxygen availability and cellular redox state [103,104]. Experimental studies in neural stem and progenitor cell models demonstrate that hypoxia and hypoxia-reoxygenation cycles induce m^6^A methylation remodeling alongside gene expression changes [105,106], supporting the concept that oxygen-related stress directly alters epitranscriptomic regulation during neurodevelopment. These findings suggest that prenatal hypoxic stress influences neurodevelopmental trajectories, at least in part, through disruption of m^6^A-dependent RNA regulation.

Maternal immune activation (MIA) is also a robust prenatal risk factor for neurodevelopmental disorders including autism spectrum disorder and schizophrenia, with documented effects on long-term gene regulatory states in the offspring’s brain [107,108]. While direct, cell-type-resolved demonstrations of m^6^A remodeling in the fetal brain following MIA remain limited, m^6^A pathways regulate innate immune and inflammatory gene expression across cell types [109,110]. m^6^A machinery responds to extracellular signaling cues in inflammatory contexts, raising the possibility that prenatal immune challenges could shape m^6^A-dependent regulation of neurodevelopmentally critical transcripts [108].

Together, these observations motivate future studies to test whether prenatal immune signaling induces persistent epitranscriptomic alterations relevant to the risk of neurodevelopmental disorders.

### 6.2. Aging and Environmental Stressors in Neurodegenerative Vulnerability

Oxidative stress is a central feature of brain aging and neurodegenerative disease, influencing RNA metabolism, protein homeostasis, and synaptic maintenance. Cellular stress states, including oxidative and metabolic stress, alter the expression or activity of m^6^A writers, erasers, and readers, thereby reshaping m^6^A landscapes [111,112]. Aging and neurodegeneration are associated with conserved reductions in global m^6^A RNA modifications, with preferential loss of synaptic transcripts involved in neuronal communication and plasticity [5]. These findings support a model in which age-related environmental and metabolic stressors converge on m^6^A-dependent regulation of synaptic gene expression, contributing to progressive synaptic vulnerability in neurodegenerative disease.

Environmental toxicants and chronic stress exposures increase neurodegenerative risk through mechanisms involving oxidative damage, inflammation, and impaired RNA processing. Although brain-specific toxicant-m^6^A studies remain relatively limited, m^6^A is characterized as a stress-responsive regulatory layer that can be perturbed by environmental insults affecting RNA stability and translation [5,56]. Altered m^6^A homeostasis may represent one molecular pathway through which environmental exposures exacerbate age-related declines in neuronal resilience and accelerate neurodegenerative processes.

### 6.3. m^6^A-Chromatin Interactions in Neural Epigenetic Regulation

m^6^A RNA methylation operates within a broader epigenetic regulatory landscape that includes DNA methylation and histone modifications. A key mechanistic link is the coupling between histone H3 lysine 36 trimethylation (H3K36me3) and m^6^A deposition. H3K36me3 promotes the co-transcriptional recruitment of the METTL3/METTL14 writer complex to actively transcribed regions [113], establishing a direct molecular connection between chromatin state and RNA methylation. Conversely, m^6^A-dependent RNA processing can influence transcriptional output, highlighting bidirectional crosstalk between RNA methylation and chromatin-based epigenetic regulation [114,115]. This interplay may be particularly relevant in the nervous system, where environmental exposures during development and aging can induce persistent changes in chromatin states that may be propagated or modulated through epitranscriptomic mechanisms. Together, these findings suggest that m^6^A may serve as an important integrator of environmental signals within multilayered epigenetic networks across the lifespan.

## 7. Discussion

This review synthesizes emerging literature on m^6^A RNA methylation across the lifespan as a central mechanism regulating brain development, plasticity, and dysregulation. Neurodevelopment and neurodegeneration have traditionally been studied separately, yet converging evidence indicates they share common epitranscriptomic bases [26,116]. By modifying RNA structure and dynamics, m^6^A fine-tunes gene expression in neurons and glia, enabling adaptive responses to diverse cellular contexts including development, stress, and environmental signals [117,118]. Dysregulation of synaptic and structural maintenance occurs when genetic variation, chronic inflammation, metabolic dysfunction, or exposure to addictive substances disrupts m^6^A homeostasis, leading to neurodevelopmental and neurodegenerative disorders.

### 7.1. Shared m^6^A Dysregulation in Neurodevelopmental and Neurodegenerative Disorders

m^6^A dysregulation in neurodevelopmental disorders manifests during early neural progenitor proliferation, synaptic determination, and dopaminergic signaling [53,119,120], contributing to core features of autism spectrum disorder, ADHD, and intellectual disability. Notably, the same m^6^A regulatory machinery (METTL3, METTL14, FTO, YTHDF proteins) is dysregulated in neurodegenerative disorders [64,119,121]. However, the consequences differ: aberrant m^6^A in neurodegeneration leads to tau hyperphosphorylation, α-synuclein dysregulation, mitochondrial dysfunction, autophagy impairment, neuroinflammation, and synaptic dysfunction [57,122]. Furthermore, chronic exposure to opiates, alcohol, and psychostimulants extensively reprograms the m^6^A epitranscriptome, amplifying maladaptive neuroplasticity through dysregulation of stress-sensitive transcripts, dopamine receptor pathways, and BDNF-antisense transcripts [123,124,125]. These findings highlight the potential role of m^6^A as an integrator of genetic risk factors, environmental influences, and stress exposures, contributing to shared epitranscriptomic alterations associated with neurodevelopmental and neurodegenerative disorders.

### 7.2. Translating m^6^A Biology into Clinical Practice

m^6^A regulatory proteins may serve as early biomarkers of disease progression. Region-specific changes in METTL3 or FTO expression precede cognitive decline in some Alzheimer’s models [60,126,127]. m^6^A-based biomarkers could enable risk stratification and personalized treatment planning in neurodevelopmental disorders. Therapeutically, m^6^A stabilization represents a promising strategy through multiple approaches: enzymatic modulation of epitranscriptomic machinery, redox and metabolic support for m^6^A restoration, or targeting m^6^A-sensitive non-coding RNAs [128,129].

Restoring m^6^A writer activity represents a compelling therapeutic strategy where m^6^A hypomethylation drives pathology. METTL3 overexpression rescued amyloid-β-induced synaptic damage, reduced tau hyperphosphorylation, and improved spatial memory in Alzheimer’s disease mouse models [61], while METTL14 restoration in substantia nigra neurons mitigated dopaminergic neuron loss and motor deficits in Parkinson’s disease models [70,71]. AAV vector-mediated delivery of METTL3 or METTL14 to defined neural populations, guided by cell-type-specific promoters, represents a precision strategy for restoring transcriptome-wide m^6^A in disorders where writer activity is specifically deficient.

FTO inhibition has emerged as a pharmacologically tractable approach for restoring m^6^A homeostasis where aberrant demethylase overactivity erases protective m^6^A marks. Several FTO inhibitors, including rhein, meclofenamic acid (MA2), and FB23-2, have been characterized biochemically and shown efficacy in preclinical models. FTO-targeted siRNA delivery via mesenchymal stem cell-derived exosomes restored m^6^A-dependent ATM mRNA stability and reduced dopaminergic neuron death in Parkinson’s disease models [75], demonstrating that biologically derived nanocarriers can achieve effective CNS delivery of m^6^A-targeting agents. ALKBH5 inhibition in microglia attenuated neuroinflammation in brain injury models [67], suggesting additional therapeutic potential in neuroinflammatory disease components. The contrasting outcomes of FTO inhibition across Huntington’s disease brain regions [78] and Parkinson’s disease dopaminergic neurons [75] underscore the necessity of region- and cell-type-targeted eraser inhibition strategies rather than global demethylase suppression. However, evidence for FTO inhibition as a therapeutic strategy in Huntington’s disease demonstrates that m^6^A modulation warrants careful, disease-specific evaluation [79].

m^6^A reader proteins offer additional therapeutic entry points. Re-expression of YTHDF1 in *Ythdf1*-knockout mice restored hippocampal long-term potentiation and rescued learning and behavioral deficits [4,26], establishing proof-of-concept for reader augmentation therapy. Disrupting pathological HNRNPA2B1-tau-m^6^A interactions may attenuate tau propagation in Alzheimer’s disease [63], while enhancing IGF2BP-mediated stabilization of neuroprotective m^6^A-marked transcripts may promote neuronal resilience under neurodegenerative stress conditions. Small molecules targeting YTH domain binding pockets, informed by structural biology, represent an emerging frontier in epitranscriptomic drug discovery.

### 7.3. Key Limitations of Current m^6^A Research

Several key limitations constrain current knowledge. First, many mechanistic studies employ rodent models or in vitro systems with limited translational relevance to human disease. Human postmortem studies, while valuable, typically have limited temporal resolution and sample scope. Second, the lack of standardized approaches for capturing m^6^A changes across development, aging, and disease hampers cross-study comparison and causal inference. Third, m^6^A’s extensive crosstalk with DNA methylation, histone modifications, and non-coding RNA systems complicates mechanistic isolation and interpretation. Fourth, expression changes in writers, erasers, and readers by bulk-tissue analysis may reflect altered cell composition (neuronal loss, gliosis) rather than regulated methylation changes. Future studies must address these limitations through improved experimental design and standardization.

### 7.4. Future Directions

Several critical research areas require development. Longitudinal studies tracking m^6^A biomarkers in at-risk populations (children at genetic risk, aging adults) before symptom onset would illuminate m^6^A’s role in disease trajectories. Integration of m^6^A profiling with single-cell and spatial epitranscriptomic technologies would enable cell-type-specific and brain-region-specific characterization of m^6^A biology. Finally, integrating m^6^A with microbiome, metabolic, and environmental variables could elucidate gene–environment interactions influencing brain health across the lifespan.

## 8. Conclusions

This review demonstrates that m^6^A RNA methylation plays a critical role in both neurodevelopmental and neurodegenerative disorders. Converging evidence indicates that m^6^A functions as a dynamic regulator of neural plasticity across the lifespan, enabling adaptive responses during development and maintenance of synaptic function during aging. Dysregulation of m^6^A regulatory machinery, including writers (METTL3/METTL14), erasers (FTO/ALKBH5), and reader proteins, contributes to aberrant neural circuit function in both neurodevelopmental and neurodegenerative disease contexts. Importantly, restoring m^6^A homeostasis through targeted modulation of these pathways represents a promising therapeutic strategy. These findings establish m^6^A dysregulation as a converging mechanism in neurological disease and position m^6^A regulators as both diagnostic biomarkers and therapeutic targets for neurodevelopmental and neurodegenerative disorders.

## Figures and Tables

**Table 1:** Association of m^6^A RNA methylation and neurodevelopmental disorders.

Neurodevelopmental Disorders	Study Design	m^6^A Machinery	Key Findings	References
Cortical neurodevelopment	Mouse (*Mettl3*/*Mettl14* depletion)	METTL3, METTL14	Mettl3/Mettl14 regulates the transition from neural progenitor proliferation to differentiation; Loss of these writers causes abnormal cortical layering and impaired neuronal differentiation.	Yoon et al., 2017 [17]
Cortical neurodevelopment	Mouse (*Ythdf2* depletion)	YTHDF2	Ythdf2 promotes degradation of methylated transcripts to refine cortical neurodevelopment and maintain proper developmental timing.	Li et al., 2018 [18]
ASD	Mouse (*Mettl3/Fto* knockout)	METTL3, FTO	Loss of Mettl3 caused abnormal cortical layering, impaired neuronal differentiation and disrupted neural circuits resembling ASD pathology.	Du et al., 2021 [19]
ASD	Cellular inflammation model	METTL3	METTL3 promotes lipopolysaccharide-induced microglia inflammation through TRAF6/NF-κB pathway.	Wen et al., 2022a [20]
ASD	Mouse (*Ythdf1* knockout)	YTHDF1	*Ythdf1*-knockout mice showed learning and memory problems.	Wen et al., 2022b [21]
ASD	Mouse (*Mettl14* depletion)	METTL14	*Mettl14* depletion in mouse embryonic nervous systems prolongs cell cycle progression of radial glia and extends cortical neurogenesis into postnatal stages.	Yen & Chen, 2021 [13]
ASD/FXS	Mouse (*Fmr1*/*Mettl14* knockout)	FMRP, METTL14	*Fmr1*/*Mettl14* knockout lead to the nuclear retention of m^6^A-modified FMRP target mRNAs that regulate neural differentiation.	Edens et al., 2019 [22]
ASD/FXS	Human U2OS/HeLa cell lines	FMRP	Depletion of nuclear FMRP isoforms promotes the accumulation of DNA bridges that are correlated with the accumulation of DNA damages and cell death.	Ledoux et al., 2023 [23]
ASD/FXS	Mouse cerebral cortex	FMRP	FMRP maintains the stability of its mRNA targets, while YTHDF2 promotes the degradation of these mRNAs.	Zhang et al., 2018 [24]
ASD	Mouse (*Ythdf1* knockout)	YTHDF1	YTHDF1-mediated translation of m^6^A-modified Robo3.1 mRNA controls pre-crossing axon guidance in spinal cord.	Zhuang et al., 2019 [25]
ASD	Mouse (*Ythdf1* knockout)	YTHDF1	*Ythdf1* knockout mice exhibited impaired learning, reduced synaptic transmission, repetitive behaviors, and striatal dysfunction that were rescued by YTHDF1 re-expression.	Liu et al., 2025 [26]
ASD	Mouse (*Ythdf1* knockout)	YTHDF1	Demonstrated impaired hippocampal synaptic transmission and learning following YTHDF1 loss.	Shi et al., 2018 [4]
ASD	Japanese population; GWAS	YTHDC2	Identified YTHDC2 as a candidate ASD susceptibility gene in East Asian populations.	Liu et al., 2016 [27]
ADHD	ADHD children; Genetic study	FTO	*FTO* SNP rs8050136 modulates the risk for ADHD.	Choudhry et al., 2013 [28]
ADHD	ADHD children; Genetic study	FTO	*FTO* minor allele at rs9939609 leads to decreased risk for symptoms of ADHD.	Velders et al., 2012 [29]
ADHD	Mouse (Lack of FTO in D2 MSNs)	FTO	Targeted manipulation of FTO altered locomotor activity, reward sensitivity, attentional performance, dopaminergic tone, neuronal excitability, and dopamine signaling pathways relevant to ADHD.	Ruud et al., 2019 [30]
ADHD	Mouse (*Fto* knockout)	FTO	FTO regulates m^6^A marks on mRNAs, controlling neuronal differentiation, synaptic formation, circuit refinement, and dopamine signaling pathways involved in attention, working memory, and executive control.	Hess et al., 2013 [31]
ADHD	Mouse hippocampus; Epitranscriptome	m^6^A modification	Reduced m^6^A levels impaired synaptic function.	Castro-Hernandez et al., 2023 [5]
ADHD	Human vmPFC; Epitranscriptome	m^6^A modification	Altered m^6^A dynamics may impair dendritic spine maturation, synaptic plasticity, and neural networks supporting attention and cognitive flexibility.	Mitsuhashi et al., 2024 [32]
ADHD	Human dlPFC; Epitranscriptome	m^6^A modification	Altered m^6^A regulation may contribute to dysfunction of attention and inhibitory-control networks.	Roy & Dwivedi, 2025 [33]
ADHD	Rat (hippocampal *Fto*)	FTO	FTO regulates synaptic and cognitive impairment by destabilizing PTEN mRNA in hypoxic–ischemic neonatal rats.	Deng et al., 2023 [34]
Intellectual Disability (ID)	Human subjects with ID	METLL5	Bi-allelic variants in *METTL5* cause autosomal recessive intellectual disability and microcephaly.	Richard et al., 2019 [35]
Intellectual Disability (ID)	Human subjects with ID	METLL5	Heterozygous loss-of-function variant in *METTL5* is associated with intellectual disability	Tao et al., 2026 [36]
Developmental delay	Zebrafish (*Ythdf2* knockout)	YTHDF2	Embryos from mothers lacking YTHDF2 (maternal mutants) exhibit developmental delay.	Zhao et al., 2017 [37]
Developmental delay	Mouse (*Ythdf2* depletion)	YTHDF2	Depletion of Ythdf2 in mice causes lethality at late embryonic developmental stages, with embryos characterized by compromised neural development.	Li et al., 2018 [18]
Rett syndrome	MeCP2-treated RPE cells	MeCP2	MeCP2 influences m^6^A modification patterns.	Zhao et al., 2023 [38]
Epilepsy	Epileptic mice	YTHDC2	YTHDC2 promotes the pathophysiology of temporal lobe epilepsy by modulating SLC7A11-dependent glutamate dysregulation in astrocytes.	Zhang et al., 2024 [39]
Epilepsy	Epileptic patients	m^6^A regulators	Identified seven significant m^6^A regulator genes in epileptic patients.	Liu et al., 2022 [40]
Epilepsy	Rat (Epilepsy model)	m^6^A regulators	Vagus nerve stimulation reduced expression of METTL3 and METTL14.	Xiong et al., 2025 [41]

ADHD: Attention-deficit/hyperactivity disorder; ASD: Autism spectrum disorder; D2 MSNs: Dopamine D2 receptor-expressing medium spiny neurons; FXS: Fragile X syndrome; GWAS: Genome-wide association study; MeCP2: methyl-CpG-binding protein 2; RPE: Retinal pigment epithelium; vmPFC: Ventromedial prefrontal cortex.

**Table 2:** Association of m^6^A RNA methylation and neurodegenerative diseases.

Neurodegenerative Diseases	Study Design	m^6^A Machinery	Key Findings	References
AD	AD mouse model; Human subjects	m^6^A level	Significantly more m^6^A sites as age increases in both mice and human.	Shafik et al., 2021 [59]
AD	Human AD hippocampus	METTL3, RBM15B	METTL3 expression was downregulated while RBM15B expression was upregulated in human AUD brain hippocampus.	Huang et al., 2020 [60]
AD	AD patient brain tissue	METTL3	Decreased neuronal m^6^A levels along with reduced expression of METTL3 in AD brains; METTL3 overexpression rescued Abeta-induced synaptic damage and cognitive impairment in vivo.	Zhao et al., 2021 [61]
AD	3xTg AD mice model (*Fto* knockout)	FTO	FTO is involved in Alzheimer’s disease by targeting TSC1-mTOR-Tau signaling.	Li et al., 2018 [62]
AD	AD subjects; AD mouse model	HNRNPA2B1	Levels of m^6^A and the m^6^A-oTau-HNRNPA2B1 complex are increased up to 5-fold in the brains of Alzheimer subjects and P301S tau mice.	Jiang et al., 2021 [63]
AD	5×FAD mouse model of AD	METTL3, WTAP	m^6^A methylation was locally reduced near amyloid plaques despite stable global brain m^6^A levels; Early METTL3 reduction preceded WTAP loss, suggesting m^6^A dysregulation is an early pathogenic event.	Gao et al., 2024 [64]
AD	Cellular inflammation model	METTL3	METTL3 is upregulated in activated microglia, promoting neuroinflammatory signaling via the TRAF6/NF-κB pathway.	Wen et al., 2022 [20]
AD	Human senescent fibroblasts	m^6^A level	m^6^A was enriched in mRNAs encoding senescence-associated secretory phenotype (SASP) factors.	Abdelmohsen et al., 2026 [65]
AD	Human microglial HMC3 cells HIV-1 infected	m^6^A level	HIV-1-induced gene expression changes may be achieved through m^6^A dysregulation, leading to cognitive disorders.	Peng et al., 2023 [66]
AD	APP/PS1 mouse model of AD	m^6^A level	m^6^A abundance is elevated in the hippocampus of 6-month-old APP/PS1 mice; m^6^A-associated immune features were involved in microglial signatures.	Qu et al., 2025 [35]
AD	Mouse cortical tissue	ALKBH5	ALKBH5 suppression in microglia/macrophages exacerbated neuroinflammation in vitro and worsened neurological deficits.	Cai et al., 2025 [67]
AD	Postmortem AD brain	m^6^A-related genes	Identified 42 differentially expressed m^6^A-related genes enriched in mitochondrial dysfunction, retinoic acid signaling, and DNA damage repair pathways.	Ni et al., 2023 [68]
AD	Human subjects	METTL3, IGF2BP3	IGF2BP3 can recognize and bind to m^6^A-methylated HDGF and VEGF mRNA, leading to increased expression and stability of HDGF and VEGF and promoting angiogenesis.	Wang et al., 2020 [69]
PD	PD mouse model (*Mettl14*-loxp)	METTL14	Deletion of Mettl14 in the substantia nigra (SN) region induced impaired motor function and locomotor activity and reduced SN mRNA m^6^A levels.	Teng et al., 2021 [70]
PD	PD mouse model (*Mettl14*-cKO)	METTL14	*Mettl14* deficiency significantly reduced ATP2A3 expression, thereby exacerbating ER homeostasis and oxidative stress, ultimately leading to DA neuronal death.	Teng et al., 2026 [71]
PD	PD rat model (brain striatum)	FTO	m^6^A reduction could induce NMDA receptor 1 expression and elevate oxidative stress and Ca(2+) influx, resulting in dopaminergic neuron apoptosis.	Chen et al., 2019 [72]
PD	PD patients; Rat PC12 cell line	HNRNPC	HNRNPC was significantly downregulated in the PD group; HNRNPC dysregulation may inhibit the proliferation of dopaminergic nerve cells, promote their apoptosis, and cause immune inflammation.	Quan et al. 2021 [73]
PD	PD mouse model	FTO	FTO downregulation reduces synaptic plasticity and causes regional neuronal damage via YTHDF3-mediated degradation of GRIN1 and GRIN3B.	Wen et al., 2024 [74]
PD	PD mouse model; MN9D cellular model	FTO	FTO promotes the expression of cellular death-related factor ataxia telangiectasia mutated (ATM) via m^6^A-dependent stabilization of ATM mRNA in dopaminergic neurons.	Geng et al., 2023 [75]
PD	PD patient transcriptome data	YTHDC2, HNRNPC, LRPPRC, IGFBP3	Identified 11 differentially expressed m^6^A-related genes; YTHDC2, HNRNPC, LRPPRC, and IGFBP3 emerged as diagnostic candidates.	Yan et al., 2024 [76]
HD	HD Hdh+/Q111 mouse model	FTO, METTL14	Impaired m^6^A remodeling in response to cognitive training associated with dysregulated FTO and METTL14 expression, linking m^6^A dysregulation to cognitive impairment in HD.	Pupak et al., 2022 [77]
HD	HD Hdh+/Q111 mouse model	FTO, METTL14	Altered hippocampal m^6^A methylation in both pre-symptomatic and symptomatic mice, with hypermethylation of Htt, Grin2b, and Ntrk2; HD mice showed impaired m^6^A remodeling during cognitive training; and hippocampal FTO knockdown restored m^6^A levels and improved memory performance.	Pupak et al., 2024 [78]
HD	R6/2 HD mouse model; human HD tissue	METTL3, TDP-43	Reduced METTL3 expression in the striatum and cortex led to decreases in nuclear m^6^A deposition; decreased m^6^A at TDP-43 motifs resulted in exon skipping in long-intron transcripts enriched for neuronal development genes.	Nguyen et al., 2025 [79]

AD: Alzheimer’s disease; PD: Parkinson’s disease; HD: Huntington’s disease.

## Data Availability

No new data were created or analyzed in this study. Data sharing is not applicable to this article.

## Abbreviations

The following abbreviations are used in this manuscript:

ATM	ATM serine/threonine kinase
AD	Alzheimer’s disease
ADHD	Attention-deficit/hyperactivity disorder
ALKBH5	α-ketoglutarate-dependent dioxygenase alkB homolog 5
ASD	Autism spectrum disorder
D2 MSNs	Dopamine D2 receptor-expressing medium spiny neurons
DAM	Disease-associated microglia
FTO	Fat mass and obesity-associated protein
FXS	Fragile X syndrome
GRIN1	Glutamate ionotropic receptor NMDA type subunit 1
GRIN2B	Glutamate ionotropic receptor NMDA type subunit 2B
GWAS	Genome-wide association study
H3K36me3	Histone H3 lysine 36 trimethylation
HD	Huntington’s disease
HNRNPA2B1	Heterogeneous nuclear ribonucleoprotein A2/B1
HNRNPC	Heterogeneous nuclear ribonucleoprotein C
ID	Intellectual disability
IGFBP3	Insulin-like growth factor-binding protein 3
m^6^A eCLIP-seq	N6-methyladenosine enhanced crosslinking and immunoprecipitation followed by sequencing
m^6^A	N^6^-methyladenosine
MAPT	Microtubule-associated protein tau
MBP	Myelin basic protein
MeCP2	Methyl-CpG-binding protein 2
MeRIP-seq	Methylated RNA immunoprecipitation sequencing
METTL14	Methyltransferase 14
METTL3	Methyltransferase 3
METTL5	Methyltransferase 5
MIA	Maternal immune activation
NTRK2	Neurotrophic receptor tyrosine kinase 2
NVU	Neurovascular unit
PD	Parkinson’s disease
PLP1	Proteolipid Protein 1
RNA-seq	RNA sequencing
RPE	Retinal pigment epithelium
TDP-43	TAR DNA-binding protein 43
vmPFC	Ventromedial prefrontal cortex
WTAP	Wilms tumor-1-associated protein
YTHDC1	YTH N^6^-Methyladenosine RNA Binding Protein C1
YTHDF1	YTH N^6^-methyladenosine RNA binding protein F1
YTHDF2	YTH N^6^-Methyladenosine RNA Binding Protein F2
YTHDF3	YTH N^6^-Methyladenosine RNA Binding Protein F3
